# Evidence-based paramedic models of care to reduce unnecessary emergency department attendance – feasibility and safety

**DOI:** 10.1186/1471-227X-13-13

**Published:** 2013-07-15

**Authors:** Judith C Finn, Daniel M Fatovich, Glenn Arendts, David Mountain, Hideo Tohira, Teresa A Williams, Peter Sprivulis, Antonio Celenza, Tony Ahern, Alexandra P Bremner, Peter Cameron, Meredith L Borland, Ian R Rogers, Ian G Jacobs

**Affiliations:** 1 Discipline of Emergency Medicine (M516), The University of Western Australia, 35 Stirling Highway, Crawley 6009, Western Australia; 2 St John Ambulance (Western Australia), 209 Great Eastern Highway, Belmont 6104, Western Australia; 3 Department of Epidemiology and Preventive Medicine, School of Public Health and Preventive Medicine, Monash University, The Alfred Centre, 99 Commercial Road, Melbourne, Victoria 3004, Australia; 4 Department of Emergency Medicine, Royal Perth Hospital, Box X2213 GPO, Perth 6847, Western Australia; 5 Emergency Department, Sir Charles Gairdner Hospital, Hospital Ave, Nedlands 6009, Western Australia; 6 Emergency Department, Fremantle Hospital, Alma Street, Fremantle 6160, Western Australia; 7 School of Population Health, The University of Western Australia, 35 Stirling Highway, Crawley 6009, Western Australia; 8 Emergency Department, Princess Margaret Hospital, GPO Box D184, Perth 6840, Western Australia; 9 School of Paediatrics and Child Health and School of Primary, Aboriginal and Rural Healthcare, University of Western Australia, Crawley 6009, Western Australia; 10 Emergency Department, St John of God Murdoch Hospital, 100 Murdoch Dr, Murdoch 6150, Western Australia; 11 Faculty of Health Sciences, Curtin University, GPO Box U1987, Perth 6845, Western Australia

**Keywords:** Pre-hospital, Extended care paramedics, Ambulance, Emergency department demand, Community care, Patient safety, Economic evaluation

## Abstract

**Background:**

As demand for Emergency Department (ED) services continues to exceed increases explained by population growth, strategies to reduce ED presentations are being explored. The concept of ambulance paramedics providing an alternative model of care to the current default ‘see and transport to ED’ has intuitive appeal and has been implemented in several locations around the world. The premise is that for certain non-critically ill patients, the Extended Care Paramedic (ECP) can either ‘see and treat’ or ‘see and refer’ to another primary or community care practitioner, rather than transport to hospital. However, there has been little rigorous investigation of which types of patients can be safely identified and managed in the community, or the impact of ECPs on ED attendance.

**Methods/Design:**

St John Ambulance Western Australia paramedics will indicate on the electronic patient care record (e-PCR) of patients attended in the Perth metropolitan area if they consider them to be suitable to be managed in the community. ‘Follow-up’ will examine these patients using ED data to determine the patient’s disposition from the ED. A clinical panel will then develop a protocol to identify those patients who can be safely managed in the community. Paramedics will then assess patients against the derived ECP protocols and identify those deemed suitable to ‘see and treat’ or ‘see and refer’. The ED disposition (and other clinical outcomes) of these ‘ECP protocol identified’ patients will enable us to assess whether it would have been appropriate to manage these patients in the community. We will also ‘track’ re-presentations to EDs within seven days of the initial presentation. This is a ‘virtual experiment’ with no direct involvement of patients or changes in clinical practice. A systems modelling approach will be used to assess the likely impact on ED crowding.

**Discussion:**

To date the efficacy, cost-effectiveness and safety of alternative community-based models of emergency care have not been rigorously investigated. This study will inform the development of ECP protocols through the identification of types of patient presentation that can be considered both safe and appropriate for paramedics to manage in the community.

## Background

Emergency Department (ED) crowding (and access block) has been described as the most serious issue currently confronting EDs [1-3]. The demand for ED services exceeds any growth that can be explained by population increase [4]. A recent Australian Institute of Health and Welfare (AIHW) report identified that “between 2009–10 and 2010–11, ED presentations increased in all states and territories, with increases ranging from 1.6% in Tasmania to 8.1% in Western Australia” [5], p vii. ED crowding has been linked to a range of adverse outcomes for patients and staff, including increased medical errors, increased patient mortality, patient dissatisfaction, high levels of work-related stress, decreased morale among ED staff and decreased capacity of EDs to respond to mass casualty incidents [2,3,6,7].

Ambulance usage is also increasing annually. In Western Australia (WA), St John Ambulance Western Australia (SJA-WA) activity in the Perth metropolitan area increased by 23% to 171,462 cases attended in the 2010/11 financial year from138,996 cases in 2006/07 [8]. For the year 2012, SJA-WA paramedic crews in metropolitan Perth attended a total of 132,862 cases and 105,327 (79.3%) were transported to ED. (“unpublished data” provided to Prof I. Jacobs by SJA-WA.) Increasing numbers of ambulance arrivals are one of the key drivers of ED demand and also increased episodes of ramping [9]. There is growing recognition that not all patients attended by paramedics actually need to be transported to ED. As part of a major overhaul of emergency services in the UK [10], the concept of ‘emergency care practitioners’ (EmCPs) emerged as an alternative model of ambulance paramedic response [10-12]. Initial reports showed that EmCPs were dealing with “54% of patients without the need for an immediate referral to another healthcare professional or emergency transportation to ED” [11]. A cluster randomised trial in the UK reported reduced ED attendance associated with Paramedic Practitioner (a similar role to EmCP) attendance, whilst maintaining patient satisfaction and safety [13].

Notwithstanding reports of the apparent success of the EmCP role in the UK, the structure of the health system, both in relation to primary care and emergency services, is different to that in both Australia and New Zealand. Extended care paramedics (ECPs) have been introduced in New Zealand [14], NSW [15] and SA [16]. In 2009 the Wellington (New Zealand) Ambulance service initiated a new model of care for a rural district with approximately 50,000 residents and a high proportion of over 65 year olds. Ambulance staff, trained in additional clinical skills, are sent to patients with conditions considered amenable to treatment in their own homes or local communities [14]. As explained, “this has shifted the focus of the ambulance service towards taking healthcare to the patient and away from automatically transporting the majority of patients to hospital” [14, p11]. A recent paper [17] reports favourable results for the first 1,000 patients attended by ECPs, with 40% of patients transported to the ED when compared to 74% of patients attended by conventional ambulance crews. Moreover, only 5% of patients who had been managed in the community by an ECP had an acute ED presentation within 7 days of that ECP attendance; although no comparison data were provided for conventional ambulance crews.

In the Sydney West-Nepean catchment area, the ECP program commenced operations in December 2007. By October 2009, a total of 22 ECPs had responded to over 10,000 cases, with a non-transport rate of 38% - 45% depending on area [15]. The South Australian Ambulance Service (SAAS) introduced an ECP programme in the metropolitan area in December 2008 [16]. A conference abstract reported that in the first 7 months “ECPs attended 1123 patients, of those 555 interventions (49.4%) were considered to have prevented an ED presentation and 60 (5.3%), were considered to have prevented a hospital admission; and no adverse events were recorded” [18].

ECPs provide alternative care pathways for patients who call for an ambulance and meet certain pre-defined criteria, such as the patient having a minor illness or injury, or only requiring basic medical advice or reassurance [15]. Through a ‘see and treat’ or a ‘see and refer’ strategy it is suggested that they can assist in reducing ambulance transport to hospital [16]. Whilst there is clear enthusiasm about the concept of ECPs as an alternative community-based model of emergency/primary health care, there is no good quality research data in Australia to support the efficacy, safety or cost-effectiveness of an ECP programme. It needs to be established that patients seen by ECPs do not end up presenting to ED within hours/days of the initial ECP attendance – possibly in a worse clinical condition than their initial presentation – or come to harm or die because of an unrecognised life-threatening condition.

Our project will develop and test (through simulation) the feasibility and safety of empirically derived clinical protocols for an extended care paramedic (ECP) role for the Perth metropolitan area. We are aware of the fact that paramedics do not have the same repertoire of clinical assessment skills as emergency physicians, nor do they have access to the same array of diagnostic tests. Of concern to all clinicians is the risk of failing to identify potentially catastrophic events, such as sepsis, stroke or myocardial infarction. Thus, while we are interested in modelling the impact of the introduction of ECPs on ED demand and ED crowding, our primary concern will be patient safety.

## Methods/Design

### Setting

Perth is located in the south-western corner of Australia. It is the capital city of WA and has a land area of 5,400 km^2^[19]. The estimated population for the Perth Statistical Division in 2011 was 1,728,867 persons [20] of whom 49.6% were men and the median age was 36 years.

### Ambulance services in WA

All road-based emergency ambulance services in WA are provided by a single ambulance service provider, St John Ambulance (Western Australia) [21]. Emergency phone calls (‘000’) for an ambulance are received by the ambulance service operations centre and computer-aided dispatch (CAD) used to assign calls to ambulance paramedic crews. During the study period, patient care data will be recorded by the paramedics using the existing electronic patient care record (e-PCR). The e-PCR data are linked to the CAD data for each episode of care.

### Emergency departments in Perth

Within the Perth metropolitan area, there are 11 Emergency Departments – 10 in public hospitals and one in a private hospital [22]. There are approximately 580,000 attendances to these EDs per annum, leading to about 170,000 admissions to hospital [23].

### Data collection

During the study period, we will include e-PCR and CAD data from SJA-WA and Emergency Department Information System (EDIS) [24] data for patients transported to the public hospital EDs [24], supplemented with hospital medical record review for a sample of patients. EDIS is a computerised patient tracking system used by the EDs in Perth to collect clinical, demographic and administrative data. Patients transported to the private hospital (without EDIS) will be manually tracked.

For the medical record review component of the study we will select a sample of patients identified as being suitable for management in the community and a research nurse will extract data from the in-hospital medical records to determine the diagnostic tests and clinical procedures performed and patient outcomes.

### Ethics approval

No direct involvement of patients is required and there will be no change in actual clinical practice in this study. Human Research Ethics Approval has been obtained from the University of Western Australia (#RA/4/1/5514), with waiver of informed consent. We also have human research ethics approval to follow-up a sample of patients at three tertiary teaching hospitals in Perth: Sir Charles Gairdner Hospital, Royal Perth Hospital, Fremantle Hospital, and the private hospital St John of God - Murdoch. This may be extended to include other hospitals depending on the findings from the initial phase of the study.

### Research plan

The study will be conducted in several consecutive phases, as follows:

### Phase 1: Collect baseline data

Over a 3 month period, we will include all patients within the Perth metropolitan area attended by SJA-WA paramedics and transported to one of the11 Perth EDs. The paramedics will routinely indicate on the e-PCR whether, in their opinion, they consider that the patient they attended could have *potentially* been managed by an ‘extended care paramedic’ through either a) ‘see and treat’ , or b) ‘see and refer’ strategies. (All paramedics will be emailed a clinical circular outlining the process for indicating on the e-PCR the possible alternative pathways.) In addition, the details of any patients attended by SJA-WA paramedics but not transported to ED (currently recorded as ‘Ambulance Not Required’ - ANR) will also be captured as part of the study.

The ED disposition and other clinical outcomes of the patients in this study cohort will be obtained via the WA EDIS data, WA hospital data and WA death registry data. Further clinical information about diagnostic tests performed and the principal reason for the patient’s admission to hospital or discharge from the ED will be abstracted from medical record reviews for a sample of patients.

The characteristics of the patients identified as potential candidates for non-transport to ED will be compared to those who were flagged as requiring transport to ED, with respect to:

a. Age, gender, socioeconomic status (based on residential address), geographical location, residential care facility, pre-hospital clinical problem code, ambulance dispatch priority code, time of day, day of week (SJA-WA data).

b. The Australasian Triage Score (ATS) [25] and presenting complaint recorded in ED.

c. ED diagnosis; ED disposition (discharge home; admit; died) and time in ED.

d. For those admitted to hospital: diagnosis, length of stay, procedures, disposition.

e. For all patients: representation at ED (or ambulance attendance) within 24 hours; 48 hours and within one week of the initial ambulance attendance.

f. A random sample of patients identified as being able to be managed in the community (proportionately across each hospital and major diagnosis category) will have medical record review by the study research nurse, to determine the nature of the clinical care received.

### Phase 2: Development of ‘trial’ ECP clinical protocols

A clinical reference group will be established with study investigators and clinical/community stakeholders (including paramedics). Based on the findings gained from Phase 1 of the study together with the best available evidence, clinical protocols for patients who could either be a) seen and treated on scene by a paramedic or b) referred to another community service will be developed. This phase of the study will involve an iterative process of refinement (through face-to-face meetings and document review) of the clinical protocols for the appropriate identification and management of patients under the extended scope of paramedic practice model of care (ECPs).

### Phase 3: Test the ECP protocols (simulation)

Following the development of clinical protocols for ECPs in Perth, a second 3-month cohort of SJA-WA patients will be studied. For this cohort, paramedics will routinely identify (on the e-PCR) whether the patient that they attended met the defined clinical protocol criteria for management by an ECP through either a) ‘see and treat’, or b) ‘see and refer’ strategies. Patient outcomes will be followed up, as described for Phase 1 of the study, to again compare the demographic, clinical characteristics, ED disposition and re-presentation at ED or readmission to hospital.

### Phase 4: Refinement of the ECP protocols

The clinical reference group will be reconvened to review the data from Phase 3 to determine the safety and appropriateness of the alternative paramedic pathway for the management of patients flagged as suitable for non-transport to ED and to refine the clinical protocols as required.

### Phase 5 – Education/training requirements

An educational reference group will review the findings from Phase 3 of the study, to determine the additional knowledge and skills that would be required for SJA-WA paramedics to undertake the required extended scope of practice. An appropriate reference group for this task will include representation from the SJA-WA College Educators, SJA-WA paramedics, University Academics, Emergency Medicine clinicians and community stakeholders.

### Phase 6: Economic evaluation

The costs of providing emergency care to patients treated in accordance with current practice (where the majority of paramedic attendances results in transport to ED), will be compared with the costs of care provided under a new model incorporating an ECP. Cost estimation will include costs incurred by the ambulance service, community health services and hospitals providing ED and inpatient care. Unit costs will be obtained from hospitals, ambulance and community services. We will initially scope the data collected in Phase 3 to assess if it is feasible to estimate the average cost of care provided for each patient contact. This would involve the use of patient level data relating to ED diagnosis, time in ED, hospital admission length of stay and procedures undertaken to calculate the average cost per patient under the current model of care (treatment provided in ED). For each contact flagged as potentially being managed by an ECP, we will estimate the cost to provide treatment using either ambulance or community services. This will allow for an estimation of the average cost of treatment under an ECP model of care. We will compare the average cost of care under current practice and the new ECP model to identify any cost savings that may offset the upfront costs of providing a training programme to the ambulance service.

### Phase 7: Systems modelling

We will also draw on the unique capacity of simulation as a systems analysis tool. Systems analysis tools are used by engineers to understand how complex systems operate, how well these systems meet operational goals, and how they can be improved [6]. They can be (and have been) used in healthcare to address a number of challenges, including ED crowding [6], but commonly have not included consideration of the pre-hospital emergency medical service [6]. EDs do not exist in isolation, but are part of a complex health system and as such policy must be based on an understanding of how they relate to pre-hospital circumstances, to the rest of the hospital and to care in the surrounding community [26], p529. Our simulation study will enable a more multi-factorial ‘system-wide’ approach to addressing ED crowding, whilst at the same time specifically enabling modelling of the impact of the introduction of ECPs on ED demand.

Using an ecological systems approach, we will model the potential impact of ECPs on ambulance service utilisation and metropolitan ED demand [27]. A whole of system approach is required to understand the complex interplay among these factors and sophisticated systems simulation can help understand the impacts of possible policy interventions and individual responses, through the running of virtual ‘what if’ experiments.

#### Statistical analysis

a) The characteristics of each of the two groups (i.e. paramedic identified potential ECP candidate patients versus non-ECP patients) will be described using percentages for categorical variables; mean ± standard deviation and median with interquartile range for continuous variables.

b) Comparisons between the two groups (i.e. ECP candidate patients versus non-ECP patients) will be performed using chi-square tests for categorical variables; and Mann-Whitney ‘U’ (non-parametric) or t-test (parametric) for continuous variables, depending on the distribution of the data. Significance will be set at p<0.05.

c) Multivariable logistic regression models will be used to (i) estimate the odds of a patient being identified by the paramedics as an ECP candidate based on their demographic and clinical condition; and (ii) model the ED disposition (admit/not admit) based on the ECP candidate status identified by the paramedics, adjusted for potential confounding characteristics of the patient and/or condition. Covariates will be included on the basis of clinical plausibility and univariate associations. Models will be run with the inclusion of all covariates deemed relevant, i.e. not using any ‘step-wise procedure’. A-priori defined interaction terms will be tested and included in the models if significant.

## Discussion

The results of our ‘virtual’ study of ECPs will provide much needed data to better inform decisions about emergency medical services in WA and other jurisdictions. Our study is congruent with the WA Department of Health primary health care principle of “implementation through consultation and evidence” [28]. Our project will bring together emergency physicians, ambulance service personnel and primary/community care providers (e.g. general practitioners and community nurses) to collaboratively develop alternative community based pathways of care for a group of patients who would otherwise be routinely (and possibly unnecessarily) transported to the ED. Collectively we will aim to develop a clinically appropriate and cost-effective alternative model of care for those patients who, despite calling for an ambulance, have health care needs that might be safely managed in the community. Reducing unnecessary ambulance transport to ED has the potential to reduce ED demand, ambulance ramping and ED crowding, as well as possibly reducing demand for in-patient services. However, we need evidence to establish that ECP models of care are safe, feasible and cost-effective.

## Abbreviations

EmCP: Emergency care practitioner; ECP: Extended care paramedic; SA: South Australia; NSW: New South Wales; WA: Western Australia; SJA-WA: St John Ambulance Western Australia.

## Competing interests

JF receives partial salary support from St John Ambulance Western Australia (SJA-WA); IJ is Clinical Services Director at SJA-WA; TA is Chief Executive Officer at SJA-WA; GA receives sitting fees for both the SJA-WA and Silver Chain Medical Policy Committees; DM is a member of the Australasian College of Emergency Medicine (ACEM) Council and Chair of the ED overcrowding sub-committee; IR receives sitting fees and is a Board member of SJA-WA. All other author(s) declare that they have no competing interests.

## Authors’ contributions

JF drafted the manuscript and all other authors provided critical review of the manuscript. HT collated and incorporated the feedback from all authors. All authors (except HT, IR & MB) were principal or associate investigators on the original funding application from the WA Department of Health – with IJ as the Chief Investigator. All authors read and approved the final manuscript.

## Pre-publication history

The pre-publication history for this paper can be accessed here:

http://www.biomedcentral.com/1471-227X/13/13/prepub
